# Successful Pregnancy following Mixed Double Embryo Transfer in a Patient with Variable Window of Implantation

**DOI:** 10.1155/2018/1687583

**Published:** 2018-05-03

**Authors:** Georgi Stamenov Stamenov, Dimitar Angelov Parvanov, Todor Angelov Chaushev

**Affiliations:** ^1^Department of Obstetrics and Gynecology, Nadezhda Women's Health Hospital, 3 “Blaga Vest” Street, Sofia, Bulgaria; ^2^Research Department, Nadezhda Women's Health Hospital, 3 “Blaga Vest” Street, Sofia, Bulgaria

## Abstract

The process of embryo implantation is carried out during the receptive stage of the endometrium in the midluteal phase of the menstrual cycle, known as window of implantation (WOI). It has been assumed that the WOI is not a constant variable in all women and the determination of its displacement is of crucial importance, especially for patients with recurrent implantation failure (RIF). Furthermore, in rare cases it could have different duration and position in the menstrual cycle even in the same woman but during different periods. Here, we report a 37-year-old woman with RIF, who was previously classified as idiopathic but has now been diagnosed as having a variable WOI. This interpretation was done after the performance of immunohistochemical and histomorphological analyses of endometrial biopsies taken in the midluteal phase during three sequential menstrual cycles. In order to solve the problem with pinpointing a variable WOI, a specific type of embryo transfer, called mixed double embryo transfer (MDET), was done where one Day 3 and one Day 5 good quality embryos were transferred simultaneously 7 days after ovulation. A viable single pregnancy was confirmed by ultrasound scan and a healthy girl was born. This case showed a unique approach in overcoming the problem in RIF patients with variable WOI.

## 1. Introduction

Successful embryo implantation could be considered as a result of the intimate communication between the embryo and maternal endometrium [1, 2]. These two worlds need to be in full synchronization in a specific time-frame, called “Window of implantation” (WOI). In this period, lasting approximately two days, a 6–8 day human embryo has a chance to be attached into the surface endometrial layer, composed of epithelial cells and to be implanted into the stromal cell layer [3].

Finding the best moment in the menstrual cycle for embryo transfer is a crucial step in overcoming the infertility problems in patients with repeated implantation failures (RIF). Displacement of the WOI during the midluteal phase occurs in at least 25% of RIF patients [4]. Some authors report even higher incidence of more than 30% out-of-phase endometrium in patients with implantation failures [5, 6]. Most of them were found to have their WOI shifted later in the cycle and the endometrium of these women was characterized as prereceptive. Changing the time of embryo transfer is a reasonable solution in these cases. However, this approach is not sufficient in those conditions where patients have variable WOI.

This case report presents a case of conception after frozen mixed double embryo transfer (MDET) of two high grade quality embryos in a patient with variable WOI. This specific type of embryo transfer includes two embryos at different developmental stage—one cleavage stage (Day 3) embryo and one blastocyst (Day 5)—that are transferred together in one frozen embryo transfer procedure in an unstimulated cycle.

To the best of our knowledge, this is the first report of an implemented mixed embryo transfer after the diagnosis of variable WOI.

## 2. Case Report

In July 2016, a 37-year-old woman attended our hospital reporting 6 consecutive unsuccessful IVF attempts and a 5-year history of primary infertility. In each IVF procedure a different number of good quality embryos were transferred and failed to implant. The infertility assessment screening showed normal condition and the case was described as idiopathic.

She had regular menstrual cycles and normal serum hormone concentrations. Her partner had normal sized testes and his semen analysis revealed normozoospermia (concentration: 110 × 10^6^; total motility: 65% and morphology according to Kruger's strict criteria: 4). Sperm DNA fragmentation index (DFI) and high DNA stainability (HDS) were also normal, below the proposed threshold value for in vitro fertility (13.4 and 14.5, resp.) [7, 8]. The peripheral karyotype of the woman and her partner were normal (46XX and 46XY, resp.).

Treatment options including classic IVF and ICSI were discussed with the couple. After consultation, the patient underwent a frozen IVF cycle, involving the transfer of two cleavage stage (Day 3) embryos in Day 5 after ovulation in natural cycle. This attempt was unsuccessful. Three months later the patient underwent a frozen IVF cycle, involving the transfer of two blastocyst stage (Day 5) embryos. This attempt did not lead to successful pregnancy again.

**Figure 1:**
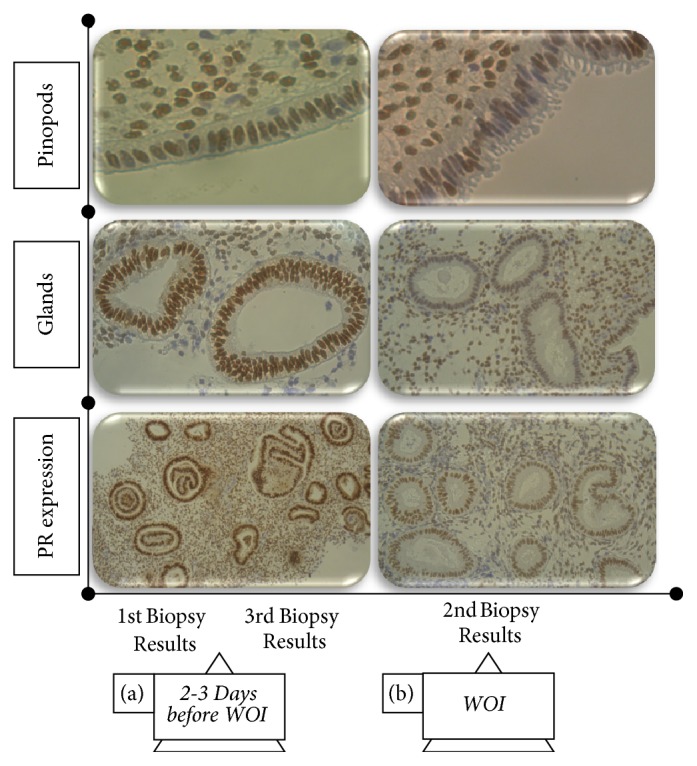
Endometrial cycle dating based on Noyes et al.'s criteria and immunohistochemical analysis for progesterone receptors (PR) of woman's endometrium. Paraffin embedded sections of the endometrium before and during the window of implantation (WOI). Pinopodes on luminal epithelium were confluent; glands secretion and stromal edema were well developed during WOI (b) compared to the prereceptive phase (a). The expression of progesterone receptors in patient's endometrium was lower during the window of implantation (b).

In order to find and pinpoint the implantation window, an endometrial biopsy was taken five days after ovulation in the midluteal phase in the natural cycle. The obtained results from histomorphological analyses, based on Noyes et al. criteria [9] and immunohistochemical analyses, revealed a three-day displacement of patient WOI and it was suggested to occur ten days after ovulation, respectively. Surprisingly, the results from the second biopsy performed one month later showed a typical WOI seven days after ovulation, which was in contradiction with the data from the first biopsy. To confirm this, a third endometrial biopsy was carried out in the next cycle but it showed the displacement of the implantation window by two days (nine days after ovulation) (Figure 1). This atypical condition that was rarely observed in other patients urged an alternative problem-solution approach.

The couple was signed for intracytoplasmic sperm injection (ICSI) treatment after stimulation with long protocol. A total of 9 oocytes were retrieved and seven of them were metaphase II. ICSI was performed and six two-pronuclear embryos were achieved. Three embryos were cultivated in a single one-step medium in Embryoscope until Day 3 and three embryos until Day 5. Embryos were vitrified by Cryotop method using Kitazato vitrification media and the Cryotop device. Both Day 3 and Day 5 embryos were thawed in the same day using Kitazato thawing media following standard protocol. After thawing embryos were cultured individually in 20 *μ*L droplets of Global Total medium under mineral oil at 37°C in 5% CO_2_ in air until embryo transfer.

The patient underwent a frozen mixed double embryo transfer (MDET) with two high grade quality embryos in a natural cycle. A natural cycle was chosen, based on previous studies that demonstrate better chance for successful implantation following the transfer of frozen-thawed embryos in natural cycles in comparison with hormone replacement therapy (HRT) cycle [10]. In addition, we took into account that the biopsies were taken in natural cycles and the obtained results about WOI would be valid only under these particular conditions. In order to exclude the potential concomitant effect of scratching procedure on the final results [11], the transfer was done four months after the last biopsy. The time of approximate ovulation was determined as the day before disappearance of the dominant follicle and it was done by dating the corpus luteum using sonographic criteria. The embryo transfer was performed seven days after ovulation. Beta hCG analysis performed on day 14 revealed 350 mIU/ml. Twenty-four days after the embryo transfer, transvaginal ultrasonography was performed and a single pregnancy was confirmed. The patient delivered a healthy girl (3650 g) by Cesarean section at 36 weeks' gestation.

## 3. Discussion

Selecting which is the most appropriate stage of embryo development for transfer is a crucial issue in IVF and it is still a matter of vigorous debate. Numerous previous reports have shown that higher pregnancy rates have been observed with blastocyst transfer (Day 5) than with transfers of early cleavage stage (Day 2 or Day 3) embryos [12–14]. On the other hand, recent discussions point out that blastocyst transfer is related to certain undesirable results, such as lower cumulative live birth rates per couple, higher risk of preterm birth, large for gestational age, monozygotic twins, and congenital anomalies, as compared to embryo transfer at cleavage stage [15–17]. However, none of the authors that have preferences to 3-day or 5-day embryo transfer did consider the possibility of applying a mixed embryo transfer.

Pinpointing the specific WOI for each patient is another key factor that plays an important role in the achievement of successful pregnancy. The available criteria for endometrial dating in order to determine WOI have been defined by Noyes and it was based on morphological variables [9]. Since then different modifications have been implemented, including gene expression analysis [4, 18] but Noyes criteria still remain the gold standard for endometrial dating. In our study, endometrial dating was carried out on endometrium on natural cycle using a set of morphological variables. In addition, an immunohistochemical assessment of progesterone receptors was done based on reports that their expression is relatively low in the midluteal phase of the menstrual cycle [19]. Determined variability in WOI in our case has led to the idea to apply mixed double embryo transfer that partially overcomes the problem with choosing the right moment for embryo transfer in а particular patient's menstrual cycle.

The implementation of this innovative strategy, which is a simultaneous transfer of two embryos at different developmental stages, combines the advantages of blastocyst and cleavage stage transfer [20]. It could be suggested that it overcomes the problems resulting from the variable WOI and should hypothetically guarantee the implantation of at least one embryo during a relatively longer period of time. Probably the transfer of several embryos at different developmental stages ensures the prolonged action of modulation factors (human chorionic gonadotropin, preimplantation factor, and granulocyte colony-stimulating factor) secreted by human embryo during its communication with the endometrium [21, 22].

To avoid a situation of no transfer at all in cases planned for blastocyst transfer, another strategy of so-called sequential or two-step transfer has been suggested by many authors [23, 24]. However, MDET has some advantages compared to the sequential embryo transfer. Firstly, it is less invasive procedure because it includes only one transfer per cycle while the sequential embryo transfer includes two consecutive transfers in the same cycle that has a possible chance of harming the transferred embryos during the second transfer. Secondly, it would be expected that MDET has a better chance to pinpoint the WOI by covering a larger period of time for implantation [20].

In summary, the applied alternative diagnostic-solution approach that includes a detection of variable WOI and the performance of mixed double embryo transfer demonstrates promising results and offers a useful tool for the management of patients with RIF.
